# The role of lysophosphatidylcholine acyltransferase 2 in osteoblastic differentiation of C2C12 cells

**DOI:** 10.1002/2211-5463.13845

**Published:** 2024-07-29

**Authors:** Shirou Tabe, Hisako Hikiji, Tomomi Hashidate‐Yoshida, Hideo Shindou, Takao Shimizu, Kazuhiro Tominaga

**Affiliations:** ^1^ Division of Oral and Maxillofacial Surgery, Department of Science of Physical Functions Kyushu Dental University Kitakyushu‐shi Japan; ^2^ School of Oral Health Sciences Kyushu Dental University Kitakyushu‐shi Japan; ^3^ Department of Lipid Life Science, Research Institute National Center for Global Health and Medicine Shinjuku‐ku Japan; ^4^ Agency for Medical Research and Development‐Core Research for Evolutional Medical Science and Technology (AMED‐CREST), AMED Chiyoda‐ku Japan; ^5^ Department of Lipidomics, Graduate School of Medicine The University of Tokyo Bunkyo‐ku Japan

**Keywords:** glycerophospholipids, lysophosphatidylcholine acyltransferase 2, lysophospholipid acyltransferase, osteoblastic differentiation

## Abstract

Glycerophospholipids, a primary component of cellular membranes, play important structural and functional roles in cells. In the remodelling pathway (Lands' cycle), the concerted actions of phospholipase As and lysophospholipid acyltransferases (LPLATs) contribute to the incorporation of diverse fatty acids in glycerophospholipids in an asymmetric manner, which differ between cell types. In this study, the role of LPLATs in osteoblastic differentiation of C2C12 cells was investigated. Gene and protein expression levels of lysophosphatidylcholine acyltransferase 2 (LPCAT2), one of the LPLATs, increased during osteoblastic differentiation in C2C12 cells. *LPCAT2* knockdown in C2C12 cells downregulated the expression of osteoblastic differentiation markers and the number and size of lipid droplets (LDs) and suppressed the phosphorylation of Smad1/5/9. In addition, *LPCAT2* knockdown inhibited Snail1 and the downstream target of Runx2 and vitamin D receptor (VDR). These results suggest that LPCAT2 modulates osteoblastic differentiation in C2C12 cells through the bone morphogenetic protein (BMP)/Smad signalling pathway.

## Graphical abstract

Protein expression of lysophosphatidylcholine acyltransferase 2 (LPCAT2, also LPLAT9), which is a phospholipid biosynthetic enzyme increased during osteoblastic differentiation in C2C12 cells. In addition, *LPCAT2* knockdown in C2C12 cells suppressed the expression of osteoblastic differentiation markers, growth of lipid droplets (LDs), and the phosphorylation of Smad1/5/9. These results suggest that LPCAT2 possibly modulates osteoblastic differentiation in C2C12 cells.

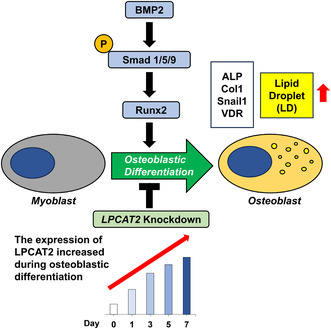

**Abbreviations**
AGPAT: 1‐acyl‐glycerol‐3‐phosphate *O‐*acyltransferase
ALP: alkaline phosphatase
ANOVA: analysis of variance
BMP: bone morphogenetic protein
BSA: bovine serum albumin
CL: cardiolipin
Col1: type 1 collagen
Col2: type 2 collagen
DMEM: Dulbecco's modified Eagle's medium
GAPDH: glyceraldehyde 3‐phosphate dehydrogenase
HRP: horseradish peroxidase
IgG: immunoglobulin G
LD: Lipid droplet
LPC: Lysophosphatidylcholine
LPCAT: lysophosphatidylcholine acyltransferase
LPLAT: lysophospholipid acyltransferase
MBOAT: membrane‐bound *O‐*acyltransferase
mRNA: messenger RNA
PAF: platelet‐activating factor
PC: phosphatidylcholine
PE: phosphatidylethanolamine
PG: phosphatidylglycerol
PI: phosphatidylinositol
PS: phosphatidylserine
RT: room temperature
Runx2: runt‐related transcription factor 2
VDR: vitamin D receptor
αMHC: alpha myosin heavy chain

Glycerophospholipids are important structural and functional components of cell membranes. In addition, they also serve as precursors of lipid mediators such as prostaglandins, leukotrienes, platelet‐activating factor (PAF), resolvin and lysophospholipids [1, 2, 3]. The contents of glycerophospholipids, such as phosphatidic acid, phosphatidylcholine (PC), phosphatidylethanolamine (PE), phosphatidylglycerol (PG), phosphatidylinositol (PI), phosphatidylserine (PS), and cardiolipin (CL), and their fatty acid composition varies depending upon the type of cell and tissue [3, 4, 5]. PC, a major mammalian phospholipid in cell membranes [6], plays a crucial role in maintaining appropriate intrinsic curvature and fluidity of membranes [7, 8, 9].

Initially, various glycerophospholipids, such as PC, PE, PG, CL, PI, and PS, are generated from glycerol‐3‐phosphate via the *de novo* pathway [10]. The glycerophospholipid acyl chains are subsequently remodelled through the remodelling pathway (Lands' cycle) [11]. In the Lands' cycle, concerted actions of phospholipases As and lysophospholipid acyltransferases (LPLATs) modify the fatty acid composition of glycerophospholipids synthesised in the *de novo* pathway to generate a mature membrane with asymmetry and diversity for cell functions and morphology [1, 4, 8, 9]. LPLATs are widely distributed in many tissues and have varying affinity for their substrates, acyl‐CoA and lysophospholipids [2, 12]. However, to date, the function of LPLATs has not been fully elucidated, although we have demonstrated that the members of LPLATs are involved in the differentiation of neurons [13] and chondrocytes [14].

Lysophospholipid acyltransferases are classified into the 1‐acyl‐glycerol‐3‐phosphate *O‐*acyltransferase (AGPAT) family and the membrane‐bound *O‐*acyltransferase (MBOAT) family [2, 12]. Lysophosphatidylcholine acyltransferases (LPCATs) are also classified into the AGPAT family and the MBOAT family. LPCAT2 (also called LPLAT9) [15] exhibits both lyso‐PAF acetyltransferase activity and LPCAT activity *in vitro* and belongs to the AGPAT family [5, 12]. LPCAT1 (also called LPLAT8) and LPCAT4 (also called LPLAT10) belong to the AGPAT family [15]. LPCAT3 (also called LPLAT12) belongs to the MBOAT family [15]. LPCAT2 converts lysophosphatidylcholine (LPC) to PC and recognises 20:4‐CoA [5, 12]. LPC is associated with the pathogenesis of various lung disorders, including acute respiratory diseases [16]. *LPCAT2* expression is high in inflammatory cells, such as macrophages and neutrophils; however, the cellular functions of this enzyme remain unknown [5, 12].

The C2C12 cell line is a subclone isolated from healthy adult C3H mouse femoral muscle, which is generally used to investigate *in vitro* differentiation of skeletal muscles [17, 18]. Lysophosphatidic acid acyltransferase 3 (LPAAT3, also called LPLAT3 and AGPAT3) [15], one of the LPLATs, is known to be involved in the maintenance of the skeletal muscle cell membranes of C2C12 cells [19]. Bone morphogenetic protein 2 (BMP2) modulates the differentiation pathway of C2C12 myoblasts to produce cells of the osteoblast lineage [20]. Therefore, C2C12 cells are commonly used to investigate osteoblastic differentiation. In this study, the role of LPLATs in osteoblastic differentiation of C2C12 cells was examined. We found that gene and protein expression levels of LPCAT2 are increased during osteoblastic differentiation. Furthermore, we found that *LPCAT2* knockdown decreased the expression of osteoblastic differentiation markers and alkaline phosphatase (ALP) activity.

Lipid droplet (LD) formation is known to be mediated by LPCAT2 [21, 22] and is involved in osteoblastic differentiation [23]. We found that LDs were present during osteoblastic differentiation of C2C12 cells. Furthermore, LP*CAT2* knockdown decreased the number and size of LDs.

Phosphorylation of Smad1/5/9 inhibits myogenic differentiation, thereby regulating the osteoblastic differentiation of C2C12 cells [24]. Snail1 is necessary for the first step of osteoblastic differentiation [25]. Knockdown of *LPCAT2* suppressed Smad1/5/9 phosphorylation. Furthermore, *LPCAT2* knockdown inhibited the expression of *Snail1*. These results suggest that LPCAT2 plays a crucial role in the osteoblastic differentiation of C2C12 cells through the BMP/Smad signalling pathway.

## Materials and methods

### Reagents

*LPCAT2* small interfering RNA (siRNA; ON‐TARGET plus mouse LPCAT2: L‐052470‐01) was purchased from Horizon Discovery (Cambridge, UK). Horseradish peroxidase (HRP)‐conjugated anti‐mouse and anti‐rabbit immunoglobulin G (IgG) antibodies were purchased from GE Healthcare (Pittsburgh, PA, USA). Anti‐glyceraldehyde 3‐phosphate dehydrogenase (GAPDH) monoclonal antibody and control siRNA (SC: 37007) were purchased from Santa Cruz Biotechnology (Santa Cruz, CA, USA). Bovine serum albumin (BSA) and 0.25% trypsin‐ethylenediaminetetraacetic acid were obtained from Sigma‐Aldrich (St. Louis, MO, USA).

### Cell culture and osteoblastic differentiation

C2C12 cells (RCB0987; Riken Bio Resource Center Cell Bank, Ibaragi, Japan) were cultured in Dulbecco's modified Eagle's medium (DMEM; Wako, Osaka, Japan) supplemented with 5% fetal bovine serum (Sigma‐Aldrich) and 1% penicillin–streptomycin mixed solution (Nacalai Tesque, Kyoto, Japan). Cells were incubated at 37 °C with 95% air and 5% CO_2_.

To induce osteoblastic differentiation [20], C2C12 cells were plated in 6‐well tissue culture plates at a density of 2.0 × 10^5^ cells per well and cultured in differentiation medium containing DMEM with 300 ng·mL^−1^ human recombinant BMP2 (R&D Systems, Minneapolis, MN, USA).

### Real‐time quantitative PCR

Total RNA was extracted from the cells using the RNeasy Mini Kit (QIAGEN, Valencia, CA, USA) according to the manufacturer's protocol. RNA was transcribed using the ReverTra Aceq qPCR RT Kit (Toyobo, Osaka, Japan) and the complementary DNA was amplified using the Program Temp Control System (Astec, Fukuoka, Japan). Quantitative PCR was performed using the LightCycler®96 System (Nippon Genetics, Tokyo, Japan) with FastStart Essential DNA Green Master (Nippon Genetics). Messenger RNA (mRNA) levels of target genes were normalised to those of GAPDH. The primers used were: LPCAT1, 5′‐GTGCACGAGCTGCGACT‐3′ (forward) and 5′‐GCTGCTCTGGCTCCTTATCA‐3′ (reverse); LPCAT2, 5′‐GTCCAGCAGACTACGATCAGTG‐3′ (forward) and 5′‐CTTATTGGATGGGTCAGCTTTTC‐3′ (reverse); LPCAT3, 5′‐TCAGGATACCTGATTTGCTTCCA‐3′ (forward) and 5′‐GGATGGGTCTGTTGCACCAAGTAG‐3′ (reverse); LPCAT4, 5′‐TTCGGTTTCAGAGGATACGACAA‐3′ (forward) and 5′‐AATGTCTGGATTGTCGGACTGAA‐3′ (reverse); ALP, 5′‐GGTATGGGCGTCTCCACAGT‐3′ (forward) and 5′‐GCCCGTGTTGTGGTGTAGCT‐3′ (reverse); Col1, 5′‐GCTTCTTTTCCTTGGGGTTC‐3′ (forward) and 5′‐GAGCGGAGAGTACTGGATCG‐3′ (reverse); Col2, 5′‐AAGTCACTGAACAACCAGATTGAGA‐3′ (forward) and 5′‐AAGTGCGAGCAGGGTTCTTG‐3′ (reverse); Runx2, 5′‐GCCGAGCTCCGAAATGC‐3′ (forward) and 5′‐AGATCGTTGAACCTGGCTACTTG‐3′ (reverse); Osterix, 5′‐GGAGACCTTGCTCGTAGATTTC‐3′ (forward) and 5′‐CAGAGAGACACCCACAGAAAC‐3′ (reverse); Sox9, 5′‐ACCCACCACTCCCAAAACC‐3′ (forward) and 5′‐CGCCCCTCTCGCTTCAG‐3′ (reverse); αMHC, 5′‐CAAGACTGTCCGGAATGCA‐3′ (forward) and 5′‐GGCTTCTTGTTGGACAGGAT‐3′ (reverse); Snail1, 5′‐AACTATAGCGAGCTGCAGGA‐3′ (forward) and 5′‐GTACCAGGAGAGAGTCCCAGA‐3′ (reverse); VDR, 5′‐GCATCCAAAAGGTCATCGGC‐3′ (forward) and 5′‐AGCGCAACATGATCACCTCA‐3′ (reverse); GAPDH, 5′‐TGACAATGAATACGGCTACAGCA‐3′ (forward) and 5′‐CTCCTGTTATTATGGGGGTCTGG‐3′ (reverse).

### ALP staining

C2C12 cells were fixed with 4% paraformaldehyde (Nacalai Tesque) for 10 min on ice and stained with the ALP stain solution (Wako) for 45 min at 37 °C.

### ALP activity assay

Total protein was extracted using the cell lysis buffer (Cell Signaling Technology, Beverly, MA, USA). Protein concentration was measured using a DC protein assay kit (Bio‐Rad, Hercules, CA, USA) with BSA as a standard. ALP activity was measured using Lab Assay ALP (Wako), according to the manufacturer's protocol, and was normalised to the total protein concentration.

### Western blot analysis

Total protein was extracted, and protein concentration was measured as described in the ALP activity assay. Equivalent amounts of total protein were resolved on 12.5% polyacrylamide gels. Separated proteins were transferred onto polyvinylidene difluoride membranes (Bio‐Rad) and blocked with Blocking One (Nacalai Tesque) for 1 h at room temperature (RT). Membranes were incubated overnight at 4 °C with the following primary antibodies: anti‐LPCAT2 (1000 : 1) [26], anti‐Smad1 (Cell Signalling Technology, Danvers, MA, USA), anti‐phosphorylated Smad1/5/9 (Cell Signalling Technology), and anti‐GAPDH (Santa Cruz). Then, the membranes were incubated with HRP‐conjugated anti‐mouse or anti‐rabbit IgG for 1 h at RT. Chemiluminescence was achieved using the enhanced chemiluminescence reagent (GE Healthcare), which was detected digitally using a Multi Imager II ChemiBox (Ieda Trading Corporation, Tokyo, Japan).

### siRNA transfection

C2C12 cells were seeded in 6‐well tissue culture plates at a density of 2.0 × 10^5^ cells per well and cultured in DMEM without antibiotics for 24 h at 37 °C. The cells were transfected with 100 nm siRNAs using Lipofectamine RNAiMAX (Thermo Fisher Scientific, Waltham, MA, USA), according to the manufacturer's instructions. Control siRNA, used as a negative control, comprised a scrambled sequence having no effect on any cellular message or function. Thereafter, the cells were incubated for another 24 h at 37 °C. Finally, prior to subsequent experiments, cells were further cultured in DMEM with 300 ng·mL^−1^ of BMP2 to induce osteoblastic differentiation at 37 °C.

### Cell viability assays: trypan blue exclusion test and WST‐8 assay

Cell viability was evaluated by performing the trypan blue dye exclusion test and WST8 assay. C2C12 cells were seeded in 6‐well tissue culture plates at a density of 2.0 × 10^5^ cells per well and cultured in DMEM without antibiotics at 37 °C. The next day, C2C12 cells were transfected with control siRNA or *LPCAT2* siRNA. Twenty‐four hours post‐transfection, cells were stained with trypan blue stain solution (Nacalai Tesque); stained cells were counted using a haemocytometer (Erma, Tokyo, Japan). The WST‐8 assay was conducted using a Cell Counting kit‐8 (Dojindo, Kumamoto, Japan), according to the manufacturer's protocol. At 24 h post‐transfection, cells were incubated with 10% WST‐8 reagent for 4 h at 37 °C. From each well, 100 μL of supernatant was transferred to a fresh 96‐well plate. Cell viability was determined by measuring the absorbance at 450 nm using an iMark™ Microplate Reader.

### LPCAT enzymatic activity assay

C2C12 cells were scraped into 1 mL of ice‐cold buffer containing 20 mm Tris‐hydrogen chloride (pH 7.4), 300 mm sucrose, and Complete Protease Inhibitor Cocktail (Roche, Basel, Switzerland). Cells were sonicated twice for 30 s each on ice, using a probe sonicator (Ohtaka Works, Tokyo, Japan). The protein solutions were collected from the supernatants and centrifuged at 800 ***g*** for 10 min. Protein concentration was measured using the Bio‐Rad Protein Assay kit (Bio‐Rad). LPCAT enzymatic assay was performed as previously described [27, 28]. Briefly, proteins (0.1 μg) were mixed with 25 μm deuterium‐labelled 16:0 LPC and 1 μm each of 16:0‐, 18:1‐, 18:2‐, 20:4‐, and 22:6‐CoA (Avanti Polar Lipids, Pelham, AL, USA) at 37 °C for 10 min.

### Measurement of PC

Lipids were extracted using the method of Bligh and Dyer [29] from proteins (1 μg) used for analysis of LPCAT enzymatic activity. PC was measured by liquid chromatography‐selected reaction monitoring/mass spectrometry (LC‐SRM‐MS), as previously described [27, 28]. A Nexera ultra‐high‐performance liquid chromatography system coupled to a triple quadrupole mass spectrometer, LCMS‐8050 (Shimadzu Corporation, Kyoto, Japan) was used. An Acquity UPLC BEH C8 column (1.7 μm, 2.1 mm × 100 mm; Waters, Milford, MA, USA) was used for the separation of PC; the column temperature was 47 °C. Solvent A was 5 mm ammonium bicarbonate (Wako), solvent B was acetonitrile (Wako), and solvent C was isopropanol (Wako). The LC solvent gradient was as follows: 0 min (solvent A = 75%/solvent B = 20%/solvent C = 5%), 20 min (20/75/5), 40 min (20/5/75), 45 min (5/5/90), 50 min (5/5/90), 55 min (75/20/5). The flow rate was 0.35 mL·min^−1^, and 5 μL of sample was applied. PC was identified by the precursor ion of *m*/*z* = 184.

### Lipid droplet analysis and morphometric analysis

Lipid droplets were visualised using the fluorescent probe Lipi‐Green (Dojindo) according to the manufacturer's protocol. Images were digitally captured in real‐time and processed using BZ‐X810 imaging software (Keyence, Osaka, Japan).

Lipid droplet analysis was assessed using the Lipid droplet kit (Dojindo). C2C12 cells were incubated in the working solution at 37 °C with 95% air and 5% CO_2_ for 2 h. The cells were measured using iMark Microplate Reader (Bio‐Rad) according to the manufacturer's protocol.

The number and size of LDs were measured in 6 random fields of the well. The average of these numbers and the diameter of the LDs are considered as the number and size of LDs per well. These measurements were performed in 3 independent wells. The area of LDs staining less than 5 μm was defined as an LD.

### Statistical analyses

All statistical analyses were performed using Microsoft Excel (Microsoft Corp., Redmond, WA, USA). Data were expressed as the mean ± standard deviation and analysed using one‐way or two‐way analysis of variance (ANOVA), followed by a *post‐hoc* test (Bonferroni's correction) for multiple comparisons. Statistical differences between two groups were determined using a two‐tailed unpaired Student's *t*‐test. A *P*‐value <0.05 was considered statistically significant.

## Results

### Gene and protein expression levels of LPCAT2 increase during the osteoblastic differentiation of C2C12 cells

**Fig. 1:**
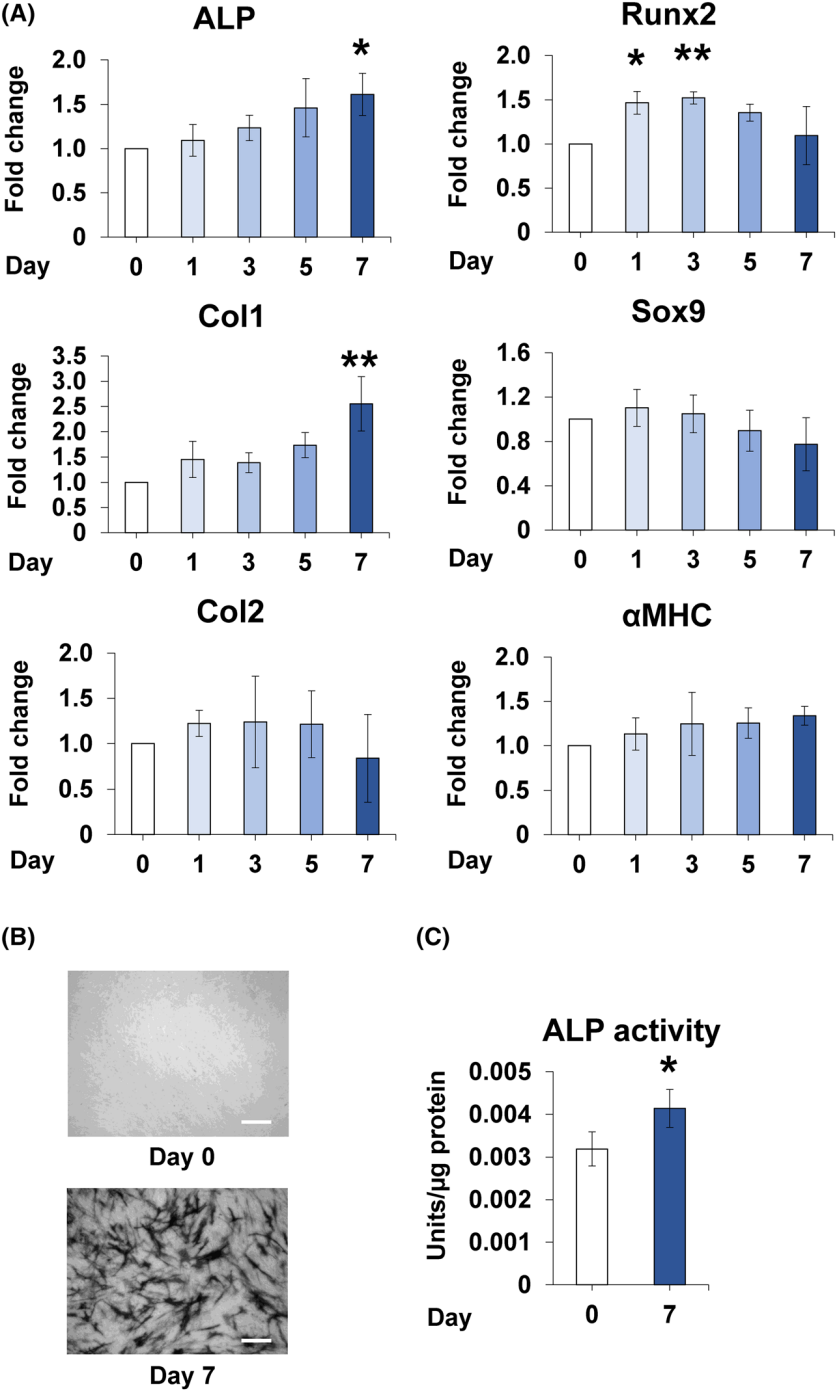
C2C12 cells differentiate into osteoblasts. (A) Gene expression of osteoblastic differentiation markers [alkaline phosphatase (*ALP*), runt‐related transcription factor 2 (*Runx*), type 1 collagen (*Col1*), and Osterix], chondrogenic markers [*Sox9* and type 2 collagen (*Col2*)], and a myogenic marker [alpha myosin heavy chain (*αMHC*)]. The fold increase in gene expression was calculated relative to day 0 (day 0 = 1) and normalised to that of glyceraldehyde 3‐phosphate dehydrogenase (GAPDH). Error bars represent mean ± standard deviation (SD), *n* = 3. *P < 0.05; ***P < 0.001, compared to day 0 with *post‐hoc* test (Bonferroni's correction) after one‐way analysis of variance. (B, C) C2C12 cells were stained using ALP staining solution, and ALP activity was measured on days 0 and 7. Scale bar is 200 μm. Error bars represent mean ± SD, *n* = 3. *P < 0.05; Student's *t*‐test was used to compare days 0 and 7.

**Fig. 2:**
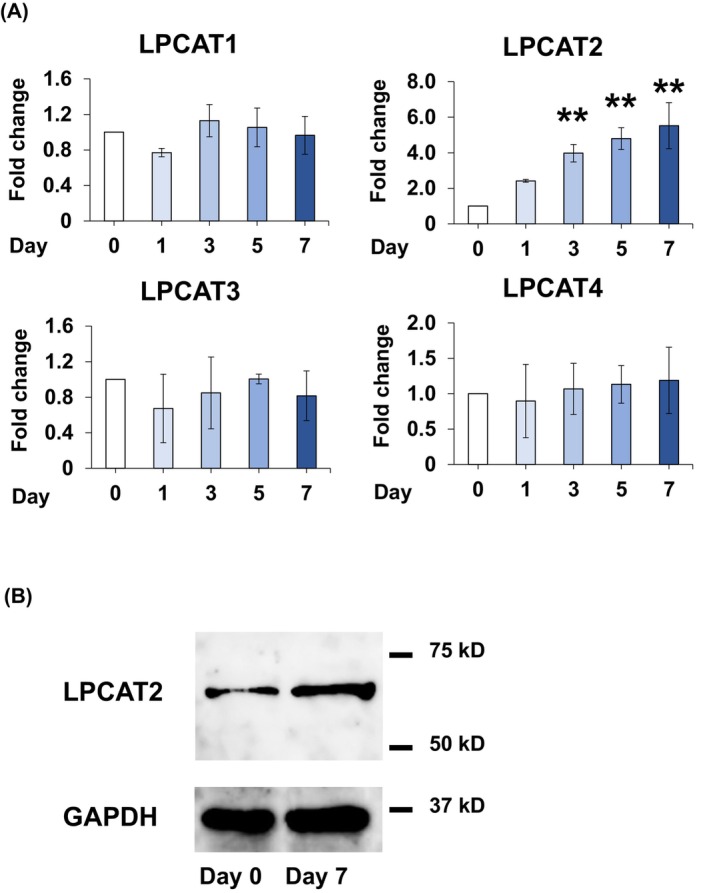
Gene and protein expression levels of lysophosphatidylcholine acyltransferase 2 (LPCAT2) increase during osteoblastic differentiation of C2C12 cells. (A) Expression of LPCATs (*LPCAT1‐4*). The fold increase in gene expression was calculated relative to day 0 [day 0, fold change = 1, normalised to that of glyceraldehyde 3‐phosphate dehydrogenase (GAPDH)]. Error bars represent mean ± standard deviation, *n* = 3. **P < 0.01, compared with day 0, *post‐hoc* test (Bonferroni's correction) after one‐way analysis of variance. (B) Protein expression of LPCAT2 and GAPDH, the internal control. [Correction added on 8 August 2024, after first online publication: Figure 2A has been updated to correct the LPCAT3 graphic bar from yellow to dark blue.]

Major osteoblastic differentiation markers include runt‐related transcription factor 2 (Runx2), ALP, type 1 collagen (Col1), and Osterix [30, 31, 32]. Expression of *Runs2* increased immediately after osteoblastic differentiation and decreased subsequently (Fig. 1A). *ALP*, *Col1*, and *Osterix* expression levels increased on day 7 of osteoblastic differentiation (Fig. 1A). Sox9 and Col2 are chondrogenic differentiation markers [30, 31], whereas alpha myosin heavy chain (αMHC) is a myogenic differentiation marker [33]. Gene expression of these markers showed no change during osteoblastic differentiation (Fig. 1A). ALP staining, used to visualise osteoblastic differentiation, demonstrated an increase in ALP levels on day 7 of osteoblastic differentiation (Fig. 1B). Furthermore, ALP activity also increased on day 7 of osteoblastic differentiation (Fig. 1C). These results confirmed the differentiation of C2C12 cells into osteoblasts. LPCAT2 levels increased both at the mRNA level and the protein levels during osteoblastic differentiation. However, no changes were observed in the expression levels of other LPLATs that were studied (Fig. 2A,B). These results suggest that LPCAT2 might be involved in osteoblastic differentiation of C2C12 cells.

### LPCAT2 knockdown inhibits osteoblastic differentiation of C2C12 cells

**Fig. 3:**
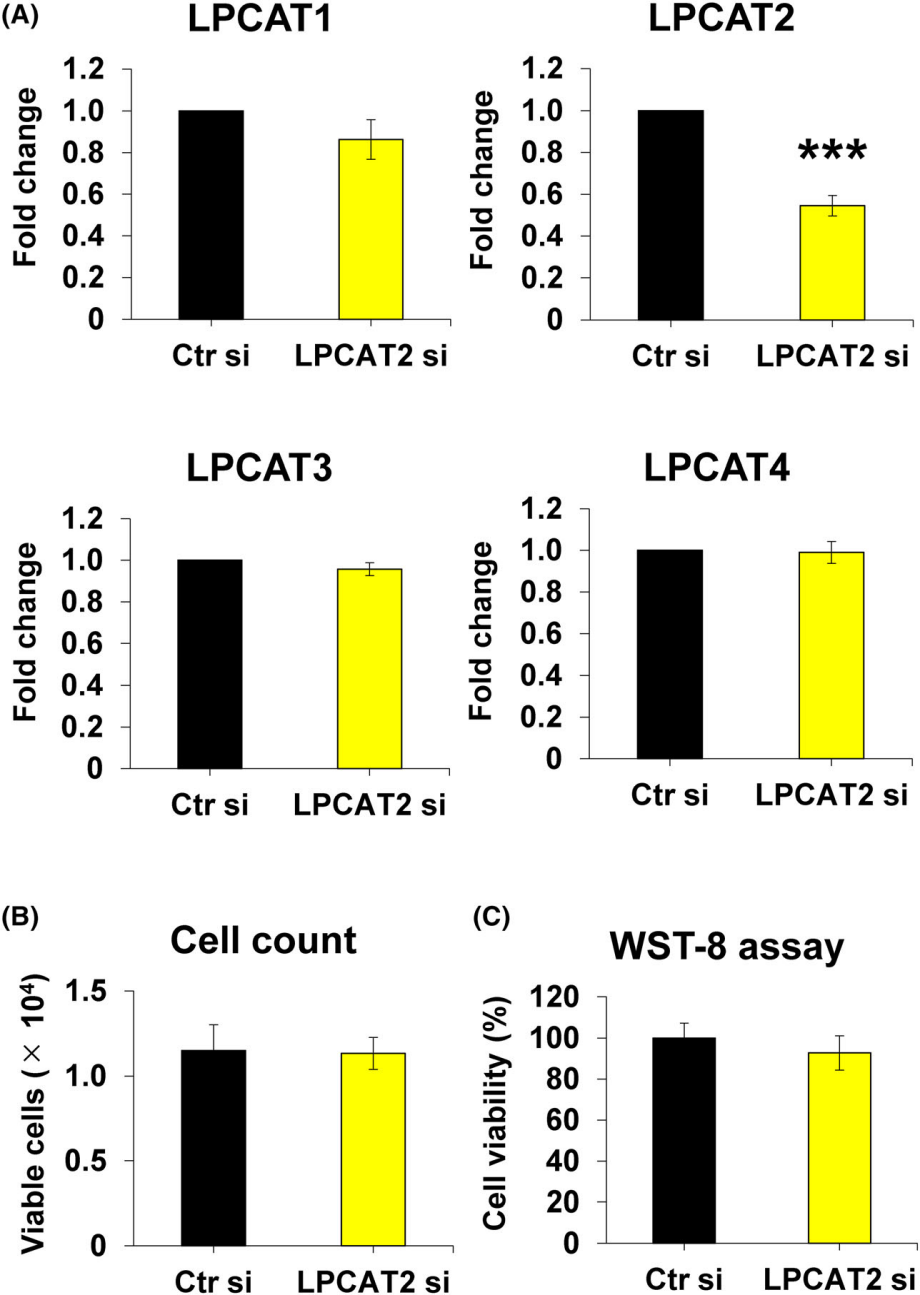
Lysophosphatidylcholine acyltransferase 2 (*LPCAT2*) knockdown decreases the gene and protein expression levels of *LPCAT2* in C2C12 cells. C2C12 cells were transfected with control small interfering RNA (Ctr si) or *LPCAT2* small interfering RNA (LPCAT2 si) for 24 h. (A) Gene expression of LPCATs (*LPCAT1‐4*) at 24 h post‐transfection. The fold increase in gene expression was calculated relative to cells transfected with Ctr si [Ctr si, fold change = 1, normalised to that of glyceraldehyde 3‐phosphate dehydrogenase (GAPDH)]. Error bars represent mean ± standard deviation (SD), *n* = 3. ***P < 0.001; Student's *t*‐test was used to compare Ctr si with LPCAT2 si. (B) Cell viability was assessed using the trypan blue dye exclusion test 24 h post‐transfection. Cells were counted in three wells, and the mean values were calculated. Error bars represent mean ± SD, *n* = 3. Student's *t*‐test was used to compare Ctr si with LPCAT2 si. (C) Cell viability was determined using the WST8 assay 24 h post‐transfection. Error bars represent mean ± SD, *n* = 3. Student's *t*‐test was used to compare Ctr si with LPCAT2 si.

**Fig. 4:**
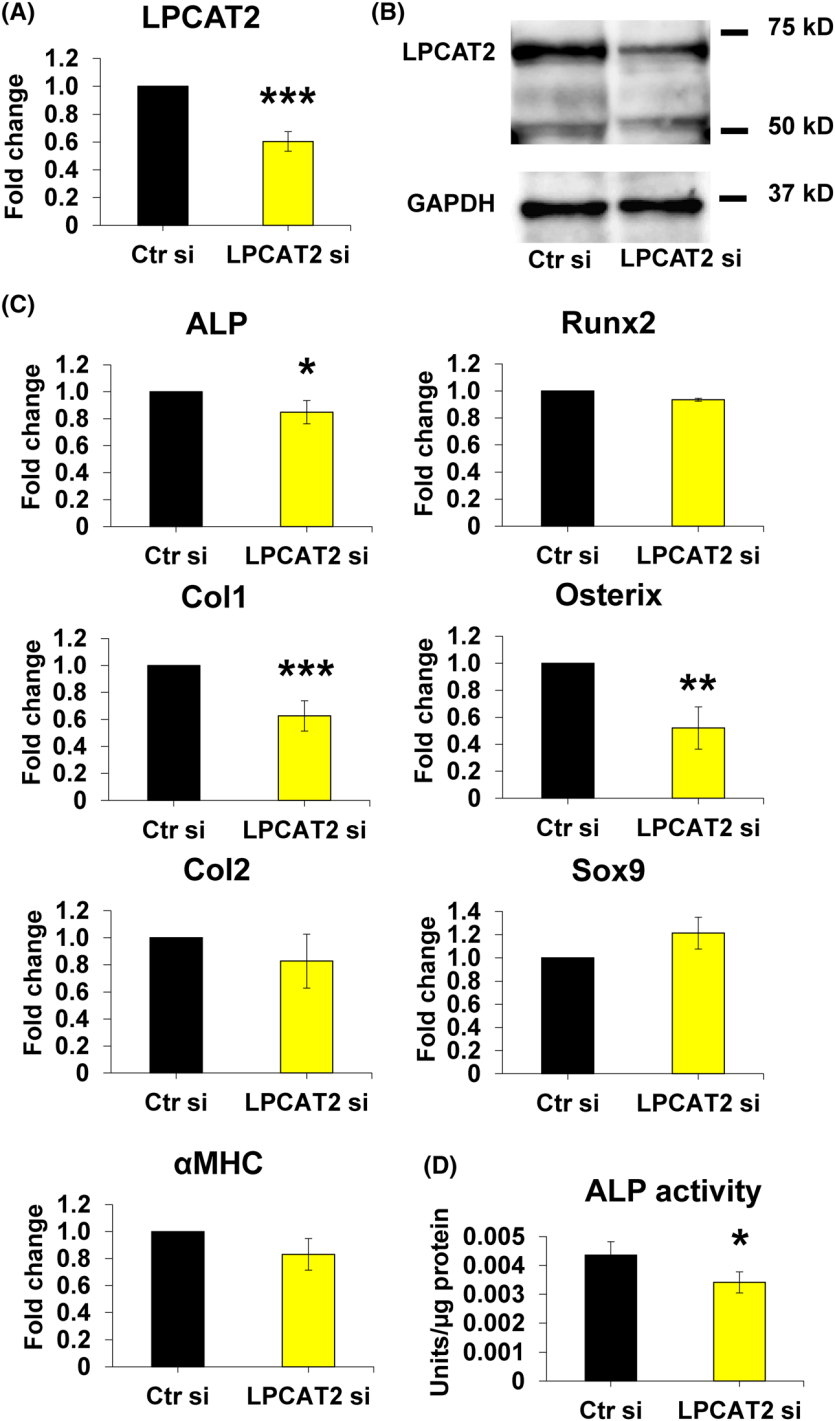
Lysophosphatidylcholine acyltransferase 2 (*LPCAT2*) knockdown inhibits osteoblastic differentiation of C2C12 cells. C2C12 cells were transfected with control small interfering RNA (Ctr si) or *LPCAT2* small interfering RNA (LPCAT2 si) for 24 h. Cells were further cultured in Dulbecco's modified Eagle's medium (DMEM) supplemented with 300 ng·mL^−1^ bone morphogenetic protein 2 (BMP2) for 7 days after transfection. (A) Gene expression of *LPCAT2*. The fold increase in gene expression was calculated relative to cells transfected with Ctr si (Ctr si, fold change = 1, normalised to that of GAPDH). Error bars represent mean ± SD, *n* = 3. ***P < 0.001, Student's *t*‐test was used to compare Ctr si with LPCAT2 si. (B) Protein expression levels of LPCAT2 and GAPDH, the internal control. (C) Gene expression of osteoblastic differentiation markers [alkaline phosphatase (*ALP*), runt‐related transcription factor 2 (*Runx2*), type 1 collagen (*Col1*), and Osterix] 7 days after transfection. The fold increase in gene expression was calculated relative to cells transfected with Ctr si [Ctr si, fold change = 1, normalised to that of glyceraldehyde 3‐phosphate dehydrogenase (GAPDH)]. Error bars represent mean ± standard deviation (SD), *n* = 3. *P < 0.05; ***P < 0.001, Student's *t*‐test was used to compare Ctr si with LPCAT2 si. (D) ALP activity on day 7 post‐transfection. Error bars represent mean ± SD, *n* = 3. *P < 0.05. Student's *t*‐test was used to compare Ctr si and LPCAT2 si. (E) Cells were further cultured in DMEM with 300 ng·mL^−1^ of BMP2 for 60 min after transfection. Protein expression levels of Smad1, phosphorylated Smad1/5/9, and GAPDH, the internal control.

*LPCAT2* knockdown decreased the expression of *LPCAT2* without affecting the transcription levels of *LPCAT1–3* (Fig. 3A) and cell viability at 24 h post‐transfection (Fig. 3B,C). *LPCAT2* knockdown also decreased the gene and protein expression of LPCAT2 on day 7 after transfection (Fig. 4A,B). *ALP* and *Col1* expression levels increased significantly on day 7 during osteoblastic differentiation of C2C12 cells (Fig. 1A). Therefore, the effect of the *LPCAT2* knockdown on osteoblastic differentiation was evaluated and reported on day 7. *LPCAT2* knockdown also decreased the expression levels of *ALP*, *Col1*, and *Osterix* (Fig. 4C). However, no change was observed in the expression levels of *Runx2* (Fig. 4C), *Sox9*, *Col2*, and *αMHC* (Fig. 4C). Furthermore, ALP activity was suppressed in *LPCAT2* knockdown cells compared to that in control cells on day 7 after transfection (Fig. 4D). These results suggest that *LPCAT2* knockdown inhibits osteoblastic differentiation of C2C12 cells.

### The number and size of LDs and LPCAT2 are correlatively induced during osteoblastic differentiation of C2C12 cells

**Fig. 5:**
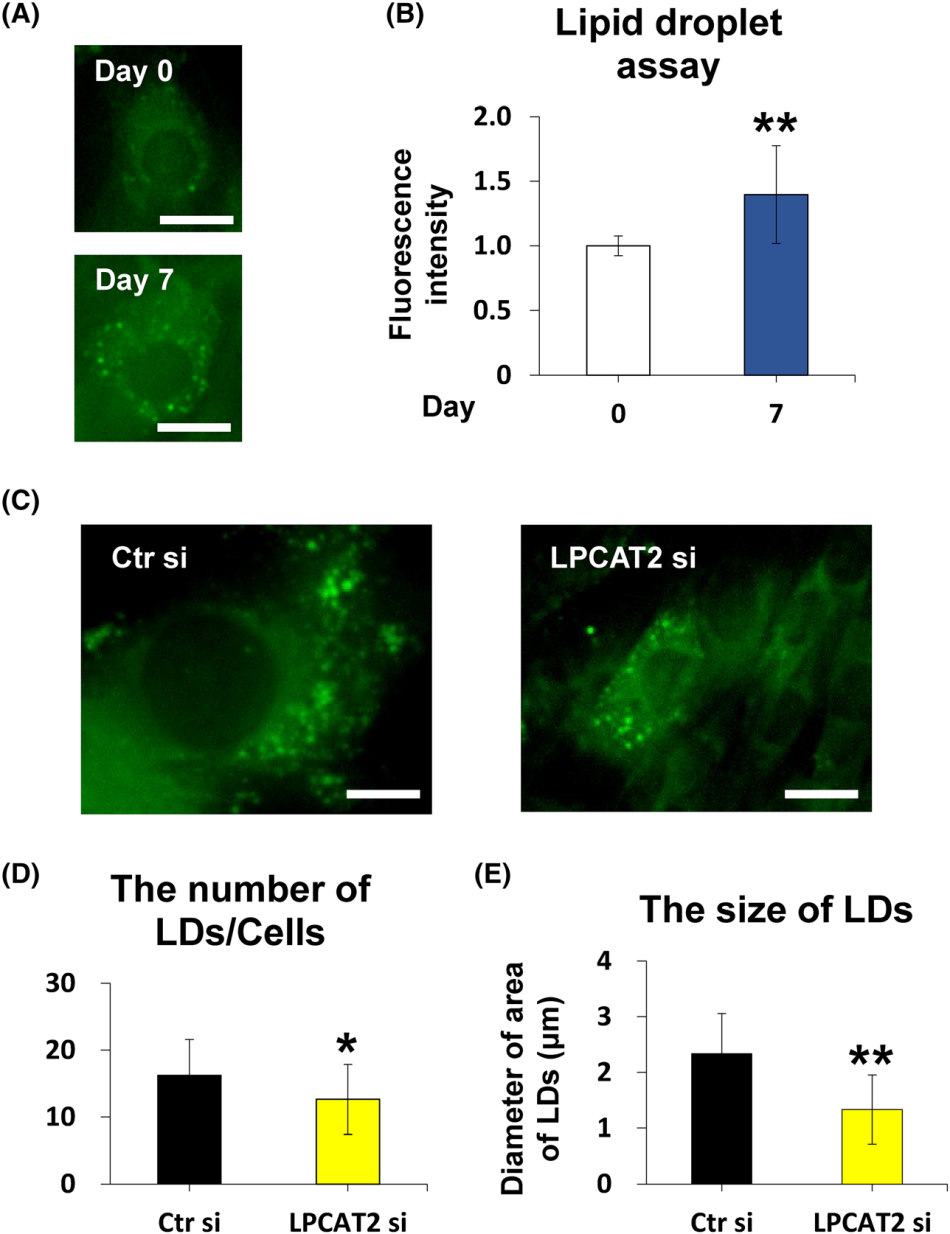
Lysophosphatidylcholine acyltransferase 2 (*LPCAT2*) knockdown inhibits osteoblastic differentiation of C2C12 cells through the BMP/Smad pathway and Snail pathway. (A) C2C12 cells differentiate into osteoblasts. Representative images of C2C12 cells were stained for LDs on days 0 and 7. (B) LDs assay was measured on days 0 and 7. Scale bar is 100 μm. Error bars represent mean ± SD, *n* = 3. **P < 0.01. Student's *t*‐test was used to compare days 0 and 7. (C) C2C12 cells were transfected with control small interfering RNA (Ctr si) or *LPCAT2* small interfering RNA (LPCAT2 si) for 24 h. Cells were further cultured in Dulbecco's modified Eagle's medium (DMEM) supplemented with 300 ng·mL^−1^ of BMP2 for 7 days after transfection. Representative images of C2C12 cells were transfected with Ctr si and LPCAT2 si and were stained for LDs. Scale bar is 20 μm. (D, E) The number and size of LDs were measured in six random fields of the well. The average number and the diameter of the LDs are considered as the number and size of LDs per well. These measurements were performed in three independent wells. The area of LDs staining more than 5 μm was defined as an LD. Error bars represent mean ± SD, *n* = 3. **P < 0.01. Student's *t*‐test was used to compare Ctr si and LPCAT2 si.

Lipid droplets are reported to play an important role in osteoblastic differentiation [21, 22], and LPCAT2 is reported to be localised to LDs [23], in addition to the endoplasmic reticulum (ER) [34]. The formation of LDs during osteoblastic differentiation of C2C12 cells was determined using Lipi‐Green and was confirmed by Lipid droplet analysis (Fig. 5A,B). *LPCAT2* knockdown significantly decreased the number and size of LDs on day 7 after transfection (Fig. 5C–E). These results suggest that LPCAT2 may play an important role in osteoblastic differentiation of C2C12 cells relating to or associated with LD production.

### LPCAT2 knockdown inhibits osteoblastic differentiation of C2C12 cells through the BMP/Smad signalling pathway

**Fig. 6:**
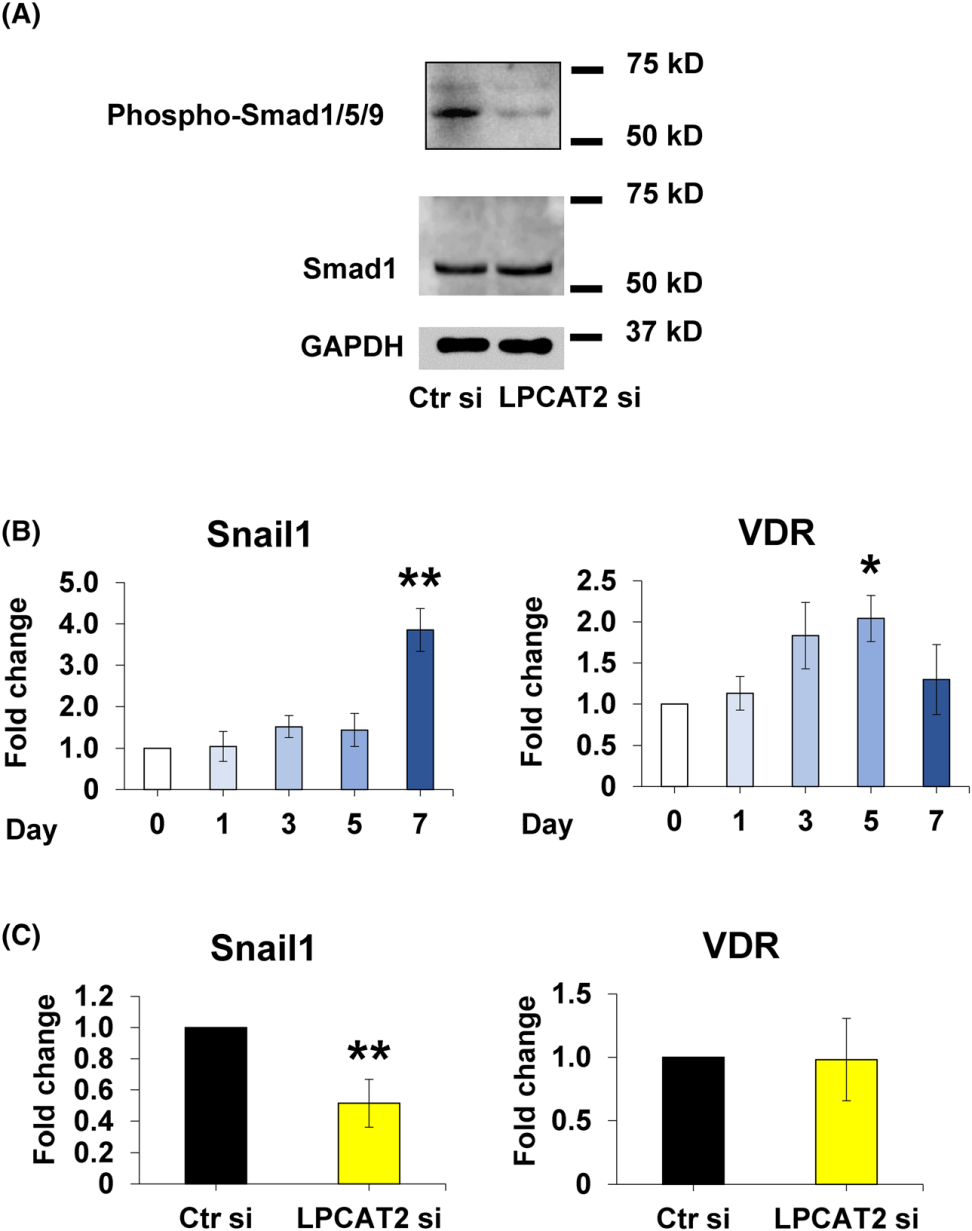
Lysophosphatidylcholine acyltransferase 2 (*LPCAT2*) knockdown inhibits osteoblastic differentiation of C2C12 cells through the BMP/Smad pathway and Snail1 pathway. (A) C2C12 cells were transfected with control small interfering RNA (Ctr si) or *LPCAT2* small interfering RNA (LPCAT2 si) for 24 h. Cells were further cultured in DMEM with 300 ng·mL^−1^ of bone morphogenetic protein 2 (BMP2) for 60 min after transfection. Protein expression levels of Smad1, phosphorylated Smad1/5/9, and GAPDH, the internal control. (B) C2C12 cells differentiate into osteoblasts. Gene expression of osteoblastic differentiation markers [Snail1 and vitamin D receptor (VDR)]. The fold increase in gene expression was calculated relative to day 0 (day 0 = 1) and normalised to that of glyceraldehyde 3‐phosphate dehydrogenase (GAPDH). Error bars represent mean ± standard deviation (SD), *n* = 3. *P < 0.05; **P < 0.01, compared to day 0 with *post‐hoc* test (Bonferroni's correction) after one‐way analysis of variance. (C) C2C12 cells were transfected with control small interfering RNA (Ctr si) or *LPCAT2* small interfering RNA (LPCAT2 si) for 24 h. Cells were further cultured in Dulbecco's modified Eagle's medium (DMEM) supplemented with 300 ng·mL^−1^ of BMP2 for 7 days after transfection. Gene expression of *LPCAT2*. Gene expression of osteoblastic differentiation markers [Snail1 and VDR] 7 days after transfection. The fold increase in gene expression was calculated relative to cells transfected with Ctr si [Ctr si, fold change = 1, normalised to that of glyceraldehyde 3‐phosphate dehydrogenase (GAPDH)]. Error bars represent mean ± standard deviation (SD), *n* = 3. **P < 0.01. Student's *t*‐test was used to compare Ctr si with LPCAT2 si.

BMP2 is known to induce osteoblastic differentiation of C2C12 cells through the BMP/Smad signalling pathway [24]. BMP/Smad signalling is initiated by the phosphorylation of Smad1/5/9, which is regulated by the binding of BMP2 to BMP receptors [24]. In order to examine the phosphorylation state, transfected cells were stimulated with BMP2 for 60 min. *LPCAT2* knockdown decreased the phosphorylation of Smad1/5/9 (Fig. 6A).

Snail1 is reported to regulate the bone mass by controlling osteoblast differentiation [35]. VDR is expressed in osteoblasts and is repressed in the presence of activated Snail1 [25]. The expression level of *Snail1* increased on day 7, while that of VDR increased gradually from day 0 to 5. However, they returned to the original levels on day 7 of osteoblastic differentiation (Fig. 6B). LPCAT2 knockdown also decreased the expression levels of Snail1. At the same time, no change was observed in the expression levels of *VDR* (Fig. 6C). These results suggest that *LPCAT2* knockdown inhibits osteoblastic differentiation of C2C12 cells by modulating the signalling pathways, including the BMP/Smad pathway and Snail1 pathway.

## Discussion

The expression pattern of each LPLAT depends on the specific cell type during cell differentiation [5]. C2C12 cells can differentiate into either myoblasts or osteoblasts. Myoblast‐like cells are remodelled from C2C12 cells, a fibroblast‐like spindle cell [17, 18]. It has been previously shown that the gene and protein expression levels of LPAAT3 increase during myoblastic differentiation in C2C12 cells [19]. In this study, gene and protein expression levels of LPCAT2 increased during osteoblastic differentiation of C2C12 cells.

Each LPLAT possesses a specific substrate preference and enzymatic activity [5, 9, 36]. Differences in substrate preference and enzymatic activity among the LPLATs produce phospholipids with a characteristic lipid composition [5, 9, 36]. LPCAT2 generates PC using LPC and 20:4‐CoA as substrates [5, 12]. Here, the fatty acid composition of PC species and LPCAT activity showed no change during osteoblastic differentiation of C2C12 cells (Figs S1 and S2). Given that the expression levels of other known LPCATs, such as *LPCAT1* (also *LPLAT8*), *LPCAT3* (also *LPLAT12*), and *LPCAT41* (also *LPLAT13*) [15], showed no change during osteoblastic differentiation of C2C12 cells (Fig. 2A), it is likely that the changes in LPCAT2 expression only have a minor contribution on global LPCAT activity and PC composition. Therefore, the global change in LPCAT enzymatic activity due to the increased gene and protein expression of LPCAT2 remains unknown; further, changes in the expression levels and activities of other enzymes that are yet to be identified might be contributing to the global change in LPCAT activity. Although other roles of LPLATs may exist, they remain unknown. Furthermore, the presence of other LPLATs may affect LPCAT activity, and it is conceivable that other enzymes may have masked the effect of the changes in the expression of LPCAT2.

It has been proposed that local arachidonate enrichment in phospholipids by LPCAT3 assists the formation of a pool of triacylglycerols required for normal triacylglycerol transfer and lipoprotein assembly in the liver and enterocytes. [27, 37]. A unique molecular species of phospholipids, 1‐oleoyl‐2‐palmitoyl PC, localised at the protrusion tip of PC12 cells after treatment with nerve growth factor [38]. It is possible that LPCAT2 contributes to changes in local lipid composition in differentiating C2C12 cells.

Previously, LPCAT2 has been reported to demonstrate lyso‐PAF acetyltransferase activity [39]. Osteoblasts showed reduced lyso‐PAF acetyltransferase activity compared to that in the osteoclasts [40]. Furthermore, osteoblasts expressed fewer PAF receptors than osteoclasts [40]. In this study, we could not detect lyso‐PAF acetyltransferase activity during osteoblastic differentiation of C2C12 cells (data not shown). Knockdown of *LPCAT2* also could not detect lyso‐PAF acyltransferase acetyltransferase activity (data not shown). These results suggest that lyso‐PAF acetyltransferase activity of LPCAT2 during osteoblastic differentiation of C2C12 cells is too weak to be detected. A previous study reported a decrease in lyso‐PAF acetyltransferase activity and the absence of PAF in an *LPCAT2* knockout mouse [41]. We need improvements in the sensitivity to measure lyso‐PAF acetyltransferase activity to elucidate potential roles of LPCAT2 in PAF production during C2C12 cell differentiation.

We previously reported that LPCAT2 was mainly localised to ER in Chinese hamster ovary‐K1 cells [34]. LPCAT2 has also been reported to be localised to lipid droplets [21, 22]. Further, LDs are reported to be important for osteoblast differentiation [23]. Our results showed that the number and size of LDs significantly increased during osteoblastic differentiation; further, the number and size of LDs decreased in *LPCAT2* knockdown cells compared to those in control cells on day 7 after transfection (Fig. 5A,B). Most phospholipids are synthesised around ER [13]. Then, these phospholipids would be transported to each specific subcellular region, including the local cellular membrane and may affect cellular morphology and function [13, 14]. LPCAT2 may also be transported to LD membranes. We could not detect the change in the fatty acid composition of PC species from total whole‐cell lysates. Despite mass spectrum data being highly reliable, liquid chromatography‐mass spectrum lacks spatial resolution. Therefore, imaging mass spectrometry may be able to show the local changes in the future.

Local PC species with different fatty acid compositions would allow the growth of LDs independent of a physical connection to the ER or other routes of PC species transport [21]. Considering the change in the number and size of LDs related to the expression of *LPCAT2*, LPCAT2 molecule may play a role in LDs locally. We could not clarify this issue from a technical point of view, so we would like to examine this issue in a future study.

The transforming growth factor‐β (TGF‐β)‐Smad3 signalling pathway regulates the expression of *LPCAT4* in mouse hepatocytes and human cells [42]. Therefore, it was presumed that the TGF‐β‐Smad3 signalling pathway might play an important role in other LPLATs. BMP2, belonging to the TGF‐β superfamily, is known to play an important role in osteoblastic differentiation through BMP/Smad signalling in C2C12 cells [19, 42]. Upon binding to the BMP receptor, BMP2 activates and phosphorylates the downstream molecules, Smad1/5/9 [43], in the BMP/SMAD signalling pathway, leading to osteoblastic differentiation. Our results showed that *LPCAT2* knockdown decreases the phosphorylation of Smad1/5/9 (Fig. 6A). These results indicate that *LPCAT2* knockdown inhibits osteoblastic differentiation through the BMP/Smad signalling pathway. LPCAT2, along with LPCAT4, might also be involved in the signalling transduction pathway.

Runx2 and Osterix are known to regulate osteoblastic differentiation [31, 43, 44]. Among them, Runx2, expressed in the early stage of osteoblastic differentiation, is downregulated during osteoblastic differentiation into mature osteoblasts [44, 45]. On the other hand, Osterix is expressed in the mature stage of osteoblast differentiation [31, 46], along with the expression of mature osteoblast genes such as *Col1* [46]. In this study, the expression of *Runx2* peaked on day 3 and subsequently decreased until day 7 (Fig. 1A). Given that the Runx2 levels were already reduced on day 7, it could explain why *LPCAT2* knockdown induced no change in the mRNA expression of *Runx2* on day 7 (Fig. 4C). In addition, *Col1* expression level increased with an increase in *Osterix* expression level during osteoblastic differentiation Since the expression of *LPCAT2* increased after day 3 in osteoblastic differentiation of C2C12 cells (Fig. 2A) and *LPCAT2* knockdown decreased the mRNA expression of *Osterix* on day 7, LPCAT2 might be associated with late stage of osteoblastic differentiation.

Snail1 is reported to regulate osteoblast differentiation and activate the expression of the early differentiation marker Col1 while it inhibits Runx2 and VDR [35]. The expression level of *Snail1* and *Col1* increased on day 7, while that of *Runx2* and *VDR* returned to the original level on day 7 during osteoblastic differentiation (Figs 1A and 6B). *LPCAT2* knockdown also decreased the expression levels of *Snail1* and *Col1*, while no change was observed in the expression levels of Runx2 and *VDR* (Figs 4C and 6C). These results suggest that *LPCAT2* knockdown inhibits osteoblastic differentiation of C2C12 cells by modulating the BMP/Smad pathway and Snail1 pathway. However, we are not exactly sure how LPCAT2 is involved in *Snail1* and lipid droplets. Therefore, we would like to examine this issue in a future research project.

In conclusion, this study reported the possible involvement of LPCAT2 in the osteoblastic differentiation of C2C12 cells.

## Conflict of interest

The authors declare no conflict of interest.

## Author contributions

ST and HH designed the experiments. TH‐Y and HS performed the LC–MS/MS analysis. TS provided professional advice on this study. ST performed the biomolecular experiments, the culture, and the staining of cells. ST, HH, HS, and KT analysed the results. ST and HH wrote the manuscript. All authors reviewed the results and the manuscript.

## Supporting information


**Fig. S1.** Lysophosphatidylcholine acyltransferase (LPCAT) activity in C2C12 cells measured with 18:2‐ and 20:4‐CoA as donors.
**Fig. S2.** Phospholipid composition of phosphocholine (PC) in C2C12 cells.

## Data Availability

The data that support the findings of this study are available in Figs 1, 2, 3, 4, 5, 6 and supplementary material of this article.
