# Supplementary material for: The role of lysophosphatidylcholine acyltransferase 2 in osteoblastic differentiation of C2C12 cells
Source: FEBS Open Bio. 2024 Jul 29;14(9):1490–502. doi: 10.1002/2211-5463.13845 (PMC11492341; doi:10.1002/2211-5463.13845)
Supplement: Supplementary file 1 — Fig. S1. Lysophosphatidylcholine acyltransferase (LPCAT) activity in C2C12 cells measured with 18:2‐ and 20:4‐CoA as donors. Fig. S2. Phospholipid composition of phosphocholine (PC) in C2C12 cells. [file FEB4-14-1490-s001.pptx]

## Slide 1
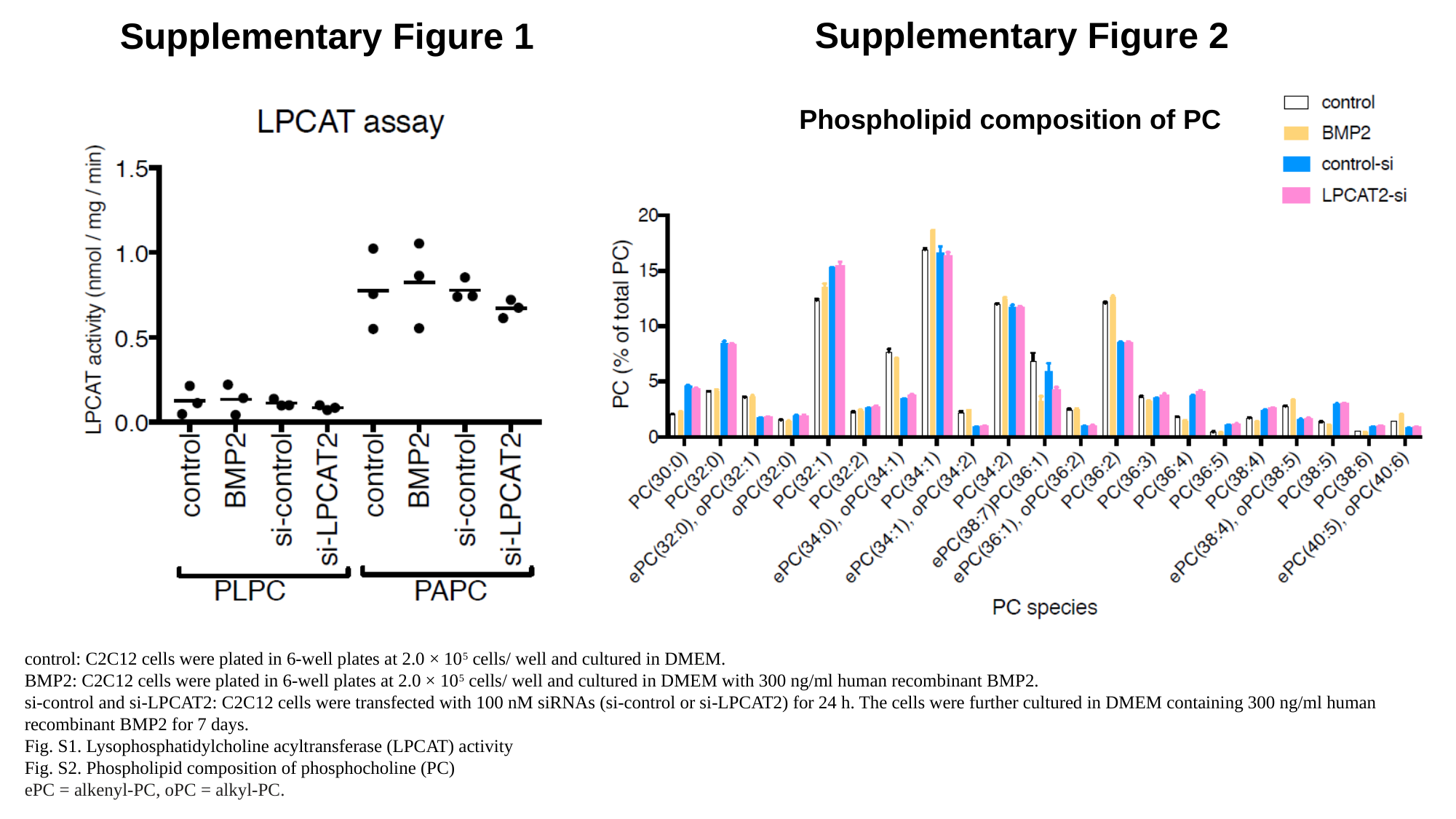

Supplementary Figure 2
Supplementary Figure 1
Phospholipid composition of PC
control: C2C12 cells were plated in 6-well plates at 2.0 × 10^5^ cells/ well and cultured in DMEM.
BMP2: C2C12 cells were plated in 6-well plates at 2.0 × 10^5^ cells/ well and cultured in DMEM with 300 ng/ml human recombinant BMP2.
si-control and si-LPCAT2: C2C12 cells were transfected with 100 nM siRNAs (si-control or si-LPCAT2) for 24 h. The cells were further cultured in DMEM containing 300 ng/ml human recombinant BMP2 for 7 days.
Fig. S1. Lysophosphatidylcholine acyltransferase (LPCAT) activity
Fig. S2. Phospholipid composition of phosphocholine (PC)
ePC = alkenyl-PC, oPC = alkyl-PC.
